# Case report: Novel *junctional sarcoplasmic reticulum protein 1* intergenic region*–anaplastic lymphoma kinase* fusion in a patient with lung adenocarcinoma responds to alectinib

**DOI:** 10.3389/fonc.2022.1019624

**Published:** 2022-10-04

**Authors:** Feng Xue, Shengyuan Xu, Cailing Jiang, Mafei Kang, Muhammad Usman, Lin Zhu

**Affiliations:** ^1^ Department of Oncology, Affiliated Hospital of Guilin Medical University, Guilin, China; ^2^ College of International Education of Guilin Medical University, Guilin, China; ^3^ Department of Radiotherapy, Affiliated Hospital of Guilin Medical University, Guilin, China

**Keywords:** intergenic region-ALK fusion, non-small cell lung cancer, alectinib, targeted therapy, case report

## Abstract

Novel *anaplastic lymphoma kinase* (*ALK*) fusions are still being discovered in non-small cell lung cancer (NSCLC). Most patients with *ALK*+ NSCLC respond favorably to *ALK* tyrosine kinase inhibitors. In this case report, we identified a novel nonreciprocal *ALK* fusion, namely, *junctional sarcoplasmic reticulum protein 1* (*JSRP1*) intergenic region–*ALK* fusion (Jintergenic: A20) *via* next-generation sequencing in a female patient initially diagnosed with stage IV B lung adenocarcinoma. Further examination of biopsy specimen and analysis of clinical samples by a multidisciplinary team confirmed the diagnosis of *ALK*+ NSCLC. At the 2- and 4-months follow-up after receiving alectinib, the patient responded rapidly, implying that alectinib had a remarkable therapeutic effect. We identified a novel JSRP1 intergenic region–*ALK* fusion as a carcinogenic mutation that responds to alectinib, thereby expanding the spectrum of *ALK* fusion partners in *ALK* + NSCLC. This study may help clinicians detect oncogenic mutations and provide timely treatment to patients with *ALK*+ NSCLC.

## Introduction

Lung cancer remains the leading cause of cancer-related deaths globally, accounting for 18% of all cancer-related deaths (1). In addition, 4–8% of patients with non-small cell lung cancer (NSCLC) harbor anaplastic lymphoma kinase (*ALK*) gene fusions, which are associated with diagnosis at a young age (2, 3). *ALK* tyrosine kinase inhibitors (TKIs) have shown remarkable effects on patients with *ALK*-rearranged NSCLC (4, 5). More than 90 distinct fusion partners have been found. Furthermore, 28 potential fusion partners due to intergenic *ALK* rearrangements have been discovered (6). *ALK* fusion is a heterogeneous biomarker that may vary in expression depending on its type (7, 8).

## Case description

**Figure 1:**
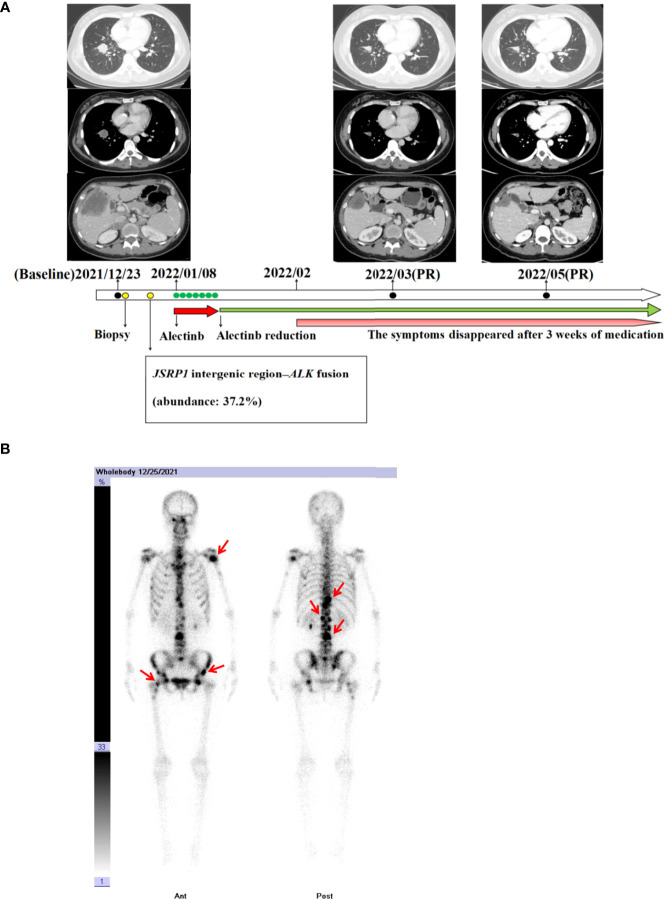
**(A)** Chest and abdomen computed tomography scans revealing the clinical response to alectinib. **(B)** Bone scan with technetium 99m-methyl diphosphonate prior to alectinib treatment revealing radioactive accumulation in the bones (red arrow).

**Figure 2:**
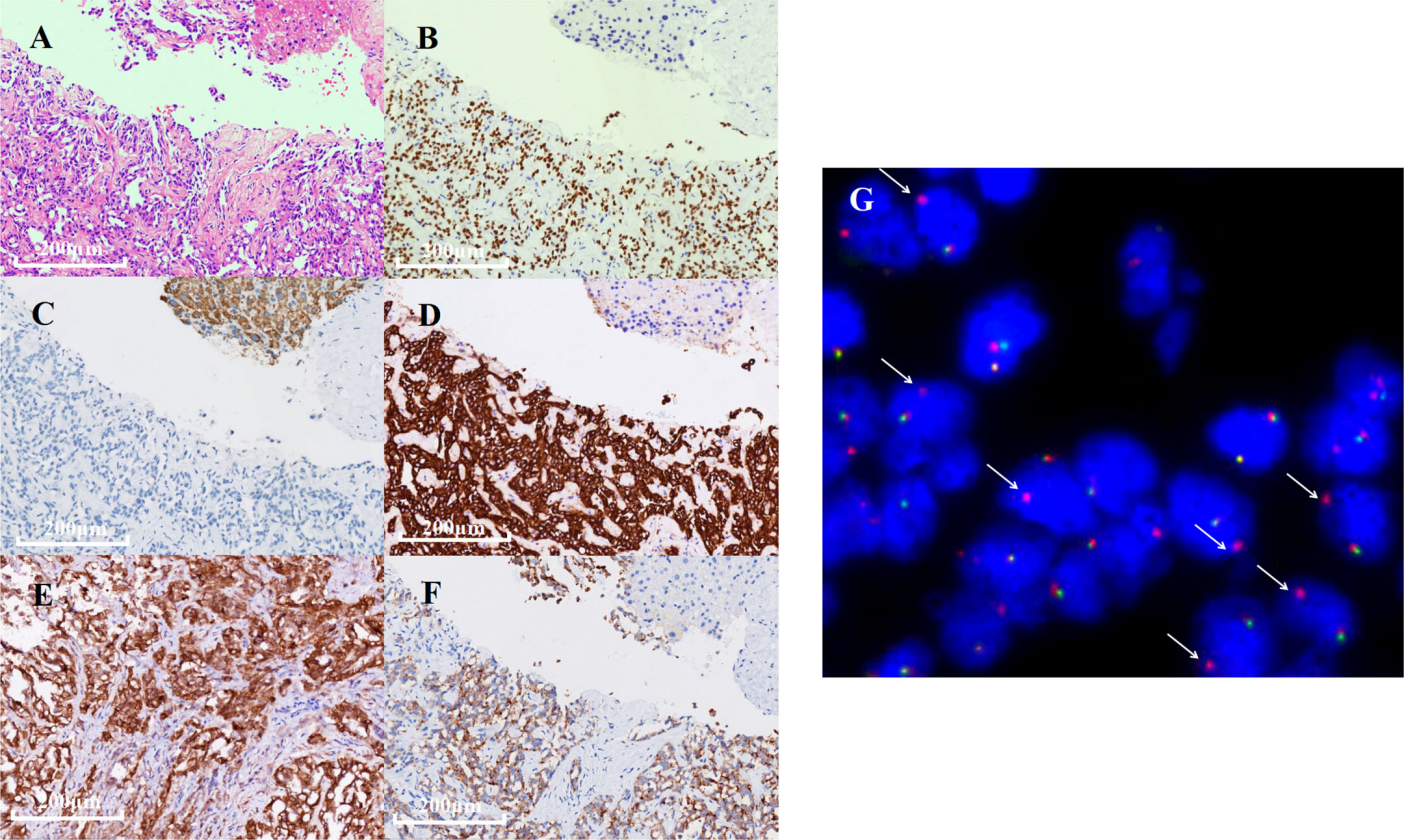
Immunohistochemistry (IHC) and fluorescence *in situ* hybridization (FISH) results of the biopsy specimen. **(A)** Hematoxylin and eosin staining revealing an adenocarcinoma. **(B–F)** IHC results of TTF-1, Hepa, CK-7, PD-L1, and ALK expression detected by D5F3 IHC assay. **(G)** FISH showed ALK gene rearrangement positive signal (one red and one yellow, indicated by white arrows).

In December 2021, a 40-year-old Chinese woman, without a family history of cancer as well as no smoking history, presented in a community hospital with upper abdominal pain for 3 months. Computed tomography (CT) scans of her abdomen revealed multiple lesions of the liver, pancreas, and bilateral adrenal gland. Chest X-ray examination revealed a lung mass in the right lower lobe. A chest CT scan revealed multiple nodules, with the largest diameter measuring 27 mm and multiple lymph node involvement around the right hilum (**Figure 1A**). The patient visited our hospital for further examination on December 22, 2021. The Eastern Cooperative Oncology Group performance status was one, and physical examination revealed no abnormal signs. Enhanced magnetic resonance imaging of the brain showed no abnormalities. Emission CT revealed abnormal radioactive concentrations in the bone, which were then diagnosed as multiple bone metastases (**Figure 1B**). At the patient’s request, ultrasound-guided liver tumor biopsy was performed on December 23, 2021. A cytologic examination of the biopsy specimen showed adenocarcinoma cells (**Figure 2A**), which tested positive for cytokeratin 7 and thyroid transcription factor-1 and negative for Hepa in immunohistochemistry (IHC) (**Figure 2B–D**). Lung adenocarcinoma with stage IV (T1bN2M1c) was confirmed by a multidisciplinary team composed of a pathologist, a thoracic radiologist, and an oncologist.

Next-generation sequencing (NGS) analysis of liver biopsy tissue was assessed by performing capture-based targeted deep sequencing using the Lung Core Panel (NextSeq 500 system, Illumina, Inc), covering the exons of 16 lung cancer-related genes (*EGFR, ALK, RET, ROS1, MET, ERBB2, KRAS, BRAF, NTRK1, NTRK2, NTRK3, CDK4, CDK6, NRAS, PIK3CA*, and *TP53*) (Guangxi Medical University REXPLO Medical Laboratory, Nanning, China). NGS revealed a novel *ALK* fusion, namely, *junctional sarcoplasmic reticulum protein 1* (*JSRP1*) intergenic region–*ALK* fusion (Jintergenic: A20) (abundance: 37.2%). The microsatellite status was microsatellite instability-stable, and the tumor proportion score of PD-L1 (antibody: 28-8) was greater than 50% (**Figure 2E**). IHC (Ventana D5F3) and fluorescence *in situ* hybridization (FISH) of the biopsy specimen were performed, which confirmed a marked *ALK* fusion at the protein and DNA levels (**Figures 2F, G**).

Most *ALK* rearrangements are sensitive to *ALK*-TKI (6). The patient was orally administered 600 mg of alectinib twice daily in January 2022. The patient complained of significant fatigue and nausea 3 days after receiving alectinib. A week after initiation, alectinib administration was reduced to 450 mg orally twice daily. The symptoms disappeared after 3 weeks of medication. At the 2- and 4-months follow-up, a CT scan revealed a remarkable reduction of the tumors in the chest, liver, pancreas, adrenal gland, and mediastinal lymph node (**Figure 1A**). Partial response was achieved in accordance with the Response Evaluation Criteria in Solid Tumors version 1.1.

Written informed consent for submitting this case report was obtained from the patient.

## Discussion

*ALK* rearrangements are common driver genes in NSCLC (9). With the increasing adoption of NGS for molecular profiling of NSCLC samples, more novel fusions and their corresponding efficacy on *ALK*-TKI have been reported (6). In this report, we identified a novel *ALK* gene fusion, *JSRP1–ALK* (Jintergenic: A20), *via* NGS and confirmed its efficacy using IHC and FISH assay. Subsequently, patient harboring this novel fusion was treated with alectinib, which exerted a remarkable effect in a short period.

**Figure 3:**
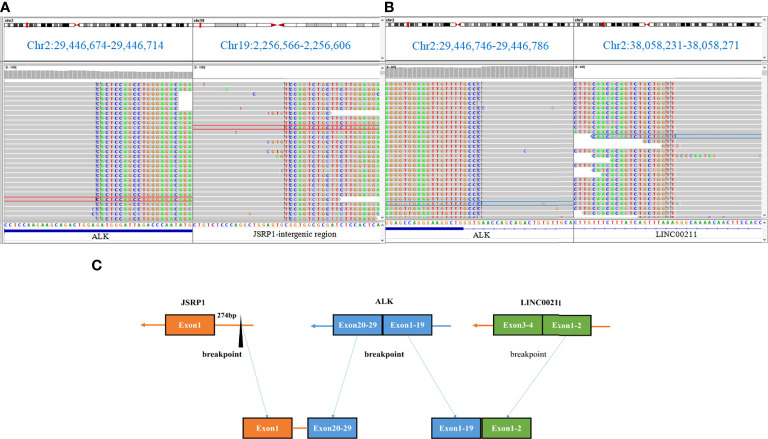
DNA-based next-generation sequencing findings for paraffin-embedded specimen of the patient. **(A, B)** The novel non-reciprocal JSRP1-ALK (Jintergenic:A20) rearrangement visualized using the Integrative Genomics Viewer.**(C)** Diagram depicting the non-reciprocal ALK fusion.

*JSRP1* is one of the proteins constituting the junctional face membrane with an apparent molecular mass of 45 kDa. It is critical in the process of functional expression of voltage-dependent Ca^2+^ channels (10, 11). In this report, *JSRP1* intergenic region rearranged to exons 20–29 of *ALK*, retaining its intact domain **(** **Figure 3A** **)**. Then, exons 1–19 of *ALK* rearranged to exon 2 of LINC00211 (A19: LINC00211) (**Figure 3B**). Based on the structure of the fusion products (**Figure 3C**), we speculated that the 3ʹ-*ALK* fusion product possesses kinase activity. Ying et al. found that uncommon *ALK* fusions detected using DNA NGS are an unreliable predictor of matched targeted therapy efficacy (12). In order to exclude the discordance of NGS, FISH, and IHC results caused by the complex biological mechanisms involved in transcriptional or post-transcriptional processes, we conducted FISH and IHC to confirm the positive *ALK* gene break and the marked *ALK* fusion protein expression in the tumor specimen. As per a previous study (13), we hypothesized that the *JSRP1* intergenic region possibly generates an N-terminal-derived product that leads to the constitutive expression of *ALK*. According to the consistency of DNA NGS, FISH, and IHC results, and the good response of the patient to alectinb, we speculated that the *JSRP1*–*ALK* (Jintergenic: A20) fusion is a carcinogenic mutation in this case. Further studies should be conducted to confirm the biological function of the *JSRP1–ALK* (Jintergenic: A20) fusion in tumorigenesis. However, the biological function of 5ʹ-*ALK* fusion translocation products remains unclear.

There are many studies and case reports on the treatment of NSCLC due to *ALK* rearrangements with crizotinib as it is the first *ALK*-TKI approved by the Food and Drug Administration for *ALK*-positive NSCLC patients. Zhang et al. reported that harboring non-reciprocal translocation is a poor predictive marker in patients with *ALK*-rearranged NSCLC treated with first-line crizotinib. Further, patients with non-reciprocal/reciprocal *ALK* translocation were reported to have a higher incidence of brain metastasis at baseline than those with classic 3ʹ-*ALK* fusion (7). Conversely, the biopsy sample had a high level of PD-L1 expression (PD-L1 TPS of > 50%). Yang et al. revealed that positive PD-L1 expression was associated with unfavorable clinical outcomes in patients with *ALK* positive lung adenocarcinoma receiving crizotinib (14). Studies have shown that different fusion modalities may have inconsistent responses to crizotinib (7, 8). Therefore, there may have not been a long survival benefit had crizotinib been given to this patient.

Compared with those receiving crizotinib, patients receiving alectinib, ceritinib, brigatinib, or lorlatinib showed better response and had considerably longer progression-free survival (PFS) in advanced *ALK*-positive NSCLC (15–18). The case we presented here had PFS for more than five months till date. Whether alectinib is more effective than crizotinb in rare *ALK* rearrangements requires more research. In addition, the resistance mechanism of rare *ALK* fusion to *ALK*-TKI is rarely reported. Further clinical observation is required to further improve the efficacy of *ALK*-TKI in patients with novel *ALK* rearrangements.

## Conclusion

We identified a novel *JSRP1* intergenic region–*ALK* fusion, expanding the spectrum of *ALK* fusion partners in patients with *ALK* + NSCLC. In addition, we found that alectinib exerted a remarkable and rapid therapeutic effect on the patient harboring this particular *ALK* fusion.

## Data availability statement

The datasets presented in this article are not readily available because of ethical/privacy restrictions. Requests to access the datasets should be directed to the corresponding author.

## Ethics statement

The studies involving human participants were reviewed and approved by ethics committee of Affiliated Hospital of Guilin Medical University. The patient provided her written informed consent to participate in this study. Written informed consent was obtained from the individual(s) for the publication of any potentially identifiable images or data included in this article.

## Author contributions

FX: Conceptualization and writing - original draft. LZ: Supervision and data curation. SX and CJ: Validation and editing of the figures. MK and MU: Writing - review and editing. All authors contributed to the article and approved the submitted version.

## Funding

This work was supported by the National Natural Science Foundation of China (Grant no. 82160471) and Beijing Xisike Clinical Oncology Research Foundation (Y-QL202101-0214).

## Acknowledgments

We would like to thank the patient and her family members. We are also grateful for Jun Lu from Shanghai Yichuang Yunkang Biotechnology for their kind assistance.

## Conflict of interest

The authors declare that the research was conducted in the absence of any commercial or financial relationships that could be construed as a potential conflict of interest.

## Publisher’s note

All claims expressed in this article are solely those of the authors and do not necessarily represent those of their affiliated organizations, or those of the publisher, the editors and the reviewers. Any product that may be evaluated in this article, or claim that may be made by its manufacturer, is not guaranteed or endorsed by the publisher.
